# Coronary risk reduction intervention for siblings and offspring of patients with premature coronary heart disease: the CRISO study protocol for a randomised controlled pilot study

**DOI:** 10.1186/s40814-021-00874-4

**Published:** 2021-08-09

**Authors:** Justin Lee Mifsud, John Stephenson, Felicity Astin, Joseph Galea

**Affiliations:** 1 Faculty of Health Sciences, University of Malta, Msida, Malta; 2 Faculty of Medicine and Surgery, University of Malta, Msida, Malta; 3 School of Human of Health Sciences, University of Huddersfield, Huddersfield, UK; 4 Research and Development, Huddersfield Royal Infirmary, Acre St, Huddersfield, UK

**Keywords:** Cardiovascular disease, Prevention, Modifiable risk, Feasibility, Acceptability, Protocol, Pilot

## Abstract

**Background:**

Research has consistently demonstrated that preventive cardiology programs have limited success, and healthy practices among high-risk individuals remain suboptimal. Furthermore, there are no current programmes in Malta that offer support to first-degree relatives of patients with premature coronary heart disease. This internal pilot study will determine the feasibility, acceptability, and potential effectiveness of a preventative intervention.

**Methods/design:**

We are conducting a 12-month single-centre, two-armed group randomised controlled trial (RCT), recruiting a sample of 100 asymptomatic first-degree relatives of patients with premature coronary heart disease (CHD). The study seeks to test an evidence-based intervention to reduce modifiable risk and determine its feasibility and acceptability. The Intervention will be delivered at an outpatient office based in a large acute academic hospital. It will comprise risk communication using an online risk calculator, a counselling style adapted from motivational interviewing, and 12 weekly telephone goal reinforcement calls (3 months). Control subjects will receive verbal lifestyle advice only. Feasibility will be assessed through recruitment and retention. Qualitative evaluation interviews will be conducted with a subsample of 24 purposefully selected participants at 12 months. Assessment for risk factor changes will be measured at pre-intervention and 6 and 12 months. Associations between variables will also be assessed descriptively.

**Discussion:**

Preventive cardiology guidelines highlighted the importance of lifestyle interventions, and lifestyle intervention adherence was proven to reduce atherosclerotic cardiovascular disease (ASCVD) risk, regardless of the individual's genetic risk. Preventive cardiology programmes may fail to adequately support persons in modifying risky behaviours, and research demonstrates that healthy practices among high-risk individuals can remain suboptimal.

Siblings and offspring of patients with premature CHD are at increased risk of ASCVD. Despite this, there is no process in place for routine screening and support to modify risk. It is hypothesised that participants assigned to the intervention arm will show more cardio-protective lifestyle-related improvement from the baseline than those in the control group. To date, this is the first trial being conducted amongst Maltese first-degree relatives. This study addresses the needed research, and the results will inform a definitive trial.

The funding institution is the University of Malta.

**Trial registration:**

ISRCTN, ISRCTN21559170; Registered 06/08/2020,

## Background and rationale

An important risk factor for atherosclerotic cardiovascular disease (ASCVD) development is family history [1]. A subset of ASCVD is coronary heart disease (CHD). The risk of having a CHD event is increased in the families of affected patients who had a premature atherosclerotic event [2], defined as an event occurring in males before 55 years and before 65 years in females [3]. In Malta, about 40% of cardiac patients will present with premature CHD [4]. First-degree relatives, such as siblings of patients with premature CHD, have an increased risk of developing the disease themselves by approximately 40%, while offspring of patients with premature CHD have about a 60% to 75% increased risk when compared with the general population [5]. Moreover, the risk is higher in males than in females [6, 7] and more substantial in middle-aged persons [8]; it increases with the number of affected relatives [5, 9] and even more so if the diseased vessel is a main left coronary artery [10].

Siblings and offspring of CHD patients are also more likely to have a higher prevalence of central obesity (≥102cm in Europid men; ≥88 cm in Europid women), hypertension, hypercholesterolemia, and smoking than people who do not have a first-degree relative with CHD [11–14]. In turn, these outcomes can concurrently act as determinants for subsequent CHD events [3, 15]; however, they can be modified or controlled. Preventive cardiology guidelines highlighted the importance of lifestyle interventions [3, 16]. Research shows that smoking cessation [17–20], adherence to a Mediterranean diet, and physical activities reduce ASCVD incidence and mortality [21–23]. Also, adherence to a Mediterranean diet and physical activity reduce risk regardless of the individual's genetic risk [24–28]. However, a gap between scientific evidence and clinical practice exists, and current prevention programmes do not support individuals with a strong family history in identifying and modifying their risk. Primary prevention is essential among individuals at increased risk [3, 16].

A recent systematic review with a meta-analysis of preventive programmes highlighted the application of counselling elements to help individuals improve their heart health risk [29]. These identified elements included: compassion, listening, affirmation, evocation, use of open questions, summarising and resolving ambivalence. Such elements could be combined with educational resources. Barrier change identification and goal setting were also identified as essential elements. It was also suggested that the application of Motivational Interviewing (MI) communication skills; open-ended questions, affirmation, reflection and summarisation (OARS), could be combined with the application of ASCVD risk scores [29]. Although a recent meta-analysis using risk scores in primary prevention efforts showed no evidence in reducing ASCVD event outcomes, this could be explained by the low event rate in individuals without pre-existent ASCVD. Therefore, it may not be feasible or practical to conduct a large trial to evaluate ASCVD risk calculators' effectiveness in reducing ASCVD outcomes [30]. However, it would be more practical to focus on modifiable risk factor change or adherence to lifestyle therapies. It is necessary to conduct further research to evaluate factors that enable and motivate individuals at risk, yet without symptomatic pre-existent ASCVD, to support them in adopting healthy lifestyle changes [29, 31].

This approach could be ideal amongst first-degree relatives of patients with premature ASCVD. Risk factor evaluation, education, and communication using counselling elements and risk scores may help establish a successful cardiovascular risk factor change.

## Aim and objectives

This study aims to evaluate a pilot study of a preventive cardiology program designed to promote risk reduction in a sample of asymptomatic first-degree relatives of patients with premature CHD.

Primary objectives are as follows:

1. Feasibility: To assess the recruitment processes and study uptake to inform the feasibility of a large-scale randomised controlled trial
2. Acceptability: To determine whether the Intervention for risk reduction is acceptable by first-degree relatives

Secondary objectives are as follows:

3. Potential effectiveness: To determine if the programme provides preliminary evidence for modifying lifestyle behaviours; physiological, anthropometric, biochemical parameters; Heart Age (estimated age based on ASCVD risk); and health literacy
4. Association between variables: To determine if the programme provides preliminary evidence for an association with lifestyle factors such as Mediterranean diet and physical activity, which themselves can act as determinants to clinical outcomes such as BMI/WC/lipids/blood pressure/HbA1c.

## Methods/design

### Setting

ASCVD risk evaluation, delivery of Intervention, and outcome measurements are being carried out from an outpatient clinic based at the Malta General State Academic Hospital.

### Study type and design

The study is an interventional pilot study. The design consists of a single-centre, 2-group pilot trial consisting of a target sample of 100 first-degree relatives. This design was chosen to determine the association with and effectiveness in addressing the modifiable cardiovascular risk factors. Qualitative evaluation interviews will be conducted with a subsample of 24 purposefully selected participants at 1 year. In this way, quantitative and qualitative data strengthen the study, allowing it to achieve all its proposed objectives [32]. More information about the study is available on a registry platform (*http://www.isrctn.com/ISRCTN21559170*).

### Materials

#### Sociodemographic questionnaire

A questionnaire was designed to collect sociodemographic data, including questions on age, sex, educational level, employment status, time of the patient coronary event, and whether participants live alone or live with others. These variables will be collected to characterise the sample. However, as randomisation is not guaranteed to result in perfectly balanced groups at baseline, variables that may be associated with the outcome will be checked for balance and controlled for in subsequent analysis if necessary.

### Primary outcomes

#### Feasibility

Feasibility will be ascertained by evaluating the recruitment and intervention processes. The study's feasibility requires that the Intervention be effectively carried out amongst an adequate number of first-degree relatives over 2 years. The assessment of the feasibility of the recruitment process will include records of the number of first-degree relatives:

- Reached via patients/ video clip (link to video clip)/ poster (link to poster)/ study participants/ clinician,
- Who expressed interest in the study,
- Who are potentially eligible, with reasons for ineligibility,
- Who agreed to participate in the trial,
- Lost to follow-up (retention and drop-out rate),

Furthermore, the assessment of the feasibility of the Intervention will include the time taken to:

- Complete the cardiovascular risk assessment,
- Deliver the Intervention (in-person session and telephone sessions).

#### Acceptability

Acceptability will be evaluated by determining how well the Intervention is received by first-degree relatives and the extent to which this Intervention could meet their needs. Post-intervention questionnaires and interviews will be used to collect this data. This requires data of the participants' most valued intervention components in terms of being supportive and the participants' views of the quality of interactions during intervention sessions, along with how well the intervention targets can be incorporated into participants' lifestyles, taking into consideration any difficulties participants might have experienced while attempting to comply with the programme goals. We will evaluate the internal reliability (i.e. internal consistency) of self-produced questionnaires during the piloting process using Cronbach’s alpha scores to identify any items that respondents are not answering consistently with their responses to other items before general implementation. We will calculate the alpha reliability value for the whole scale and will also infer individual item reliability via alpha values calculated for the scale with each item deleted in turn. Any items identified as detracting from overall reliability will be considered for removal or re-wording and re-piloted if necessary until satisfactory scale reliability is achieved.

### Secondary outcomes

#### Potential intervention effectiveness

Preliminary evidence for the effect of the intervention will be determined by an increase in the mean smoking cessation attempts, increased mean Mediterranean diet score, and physical activity level. A decrease in the mean serum lipids, blood pressure level, waist circumference, and body mass index, and Heart Age will be considered as improvements in cardiovascular risk factors if statistically significant. Any trends identified across variable results will be explored.

The following are secondary outcome measurements considered:

- Smoking status

Smoking status will be answered by a 'yes' or 'no.' As self-reporting depends on participants' honesty, those who report that they have stopped smoking will be tested for expired carbon monoxide using a carbon monoxide monitor.

- Mediterranean diet score

The 14-point Mediterranean Diet Adherence Screener (MEDAS) [33] is a fourteen-item questionnaire that assesses adherence to the Mediterranean diet with two 'yes-or-no' questions on food intake habits and another twelve questions on food consumption frequency. The questionnaire is based on the nine-item index used in the PREDIMED study, and it covers the use of Mediterranean diet habits and frequency of consumption of Mediterranean food items. If the item/question is not met, a score of zero will be recorded. Therefore, the final score will range from 0 to 14. The higher the score, the greater the adherence to the Mediterranean diet.

- Physical activity level

The rapid assessment of physical activity (RAPA) questionnaire is a 9-item valid questionnaire, which requires 2 to 5 min to complete [34]. The questionnaire is based on the Centres for Disease Control and Prevention (CDC) guidelines, suggesting a minimum of 30 min of moderate physical activity daily. The questionnaire covers a range of physical activity levels, from sedentary to regular vigorous physical activity and strength training and flexibility. The total score of the first seven items is one score for each and, thus, scoring is from 0 to 7 points. Responses to strength training and flexibility items are scored separately, with strength training = 1 point, flexibility = 2 points, or both = 3 points. Therefore, the final score would range from 0 to 10.

- Biochemical measures

Blood biochemicals (lipids, HbA1c) will be analysed using the Roche COBAS analyser, while TFT will be analysed using the Siemens ADVIA Centaur analyser. Both analysers are closed systems, and the kits that will be used are only those provided by Roche and Siemens, respectively.

- Physiological measures

Resting physiological measurements will be measured using an Omron™ blood pressure monitor, measured two to three times by auscultation, and an average will be taken as the final measurement. Heart rate shall be measured manually using the radial pulse. For both measurements, the first measurement shall be monitored after a 10-min seated rest, repeated 10 min after, and then again at the end of the session. Blood pressure will be measured in both arms.

- Anthropometric measures

Anthropometric measurements will be measured using a SECA scale with a height gauge. Numerous steps will be taken to avoid measurement errors. Weight will be taken to the nearest 0.1 kg and both measurements recorded twice. Recording of the waist circumference will be recorded while the participant is standing. A non-elastic measuring tape will be placed at the midpoint between the iliac crest and the lowest rib and at the umbilicus level. This measurement will be taken at the end of expiration.

- Heart Age

Heart Age will be calculated after obtaining the following parameters: age, sex, systolic blood pressure, cholesterol, smoking status, weight, and height. The parameter data obtained will be inputted into the online calculator to generate a score. This score will allow for the observation of changes in global risk [35].

- Health literacy

The Health Literacy Questionnaire (HLQ) has nine scales in total [36, 37]. We decided to select those scales which we think are relevant to the Intervention. The questions will not be amended as they are validated in the way they are. However, the researcher shall tell the participant that the questions asked are about their heart health.

The scales selected are: scale 1, Feeling understood and supported by healthcare professionals; scale 2, Having sufficient information to manage health; scale 3, Actively managing health.

The selected scales' response options range from 1 to 4, with the scoring as follows: 'strongly disagree’ = 1, 'disagree’ = 2, 'agree' = 3, 'strongly agree’ = 4. The total score is calculated for all the questions on that scale and divided by the number of items.

### Participants

The study population will consist of first-degree relatives of patients with premature CHD (males ≤ 55, females ≤ 65), aged 30 years and older. Males and females of Maltese ethnicity seeking cardiovascular risk assessment with no history of cardiovascular disease will be recruited for the study. Other eligibility criteria also apply:

- Participants must not have diabetes.
- Participants must not have a history of rheumatoid arthritis and chronic kidney disease.
- Participants must not have contraindications against physical activities and can go up a flight of stairs comfortably.
- Participants must not be pregnant.

### Clinician to deliver the Intervention

The clinician to deliver the Intervention is a qualified nurse who received training from Imperial College London in preventive cardiology (JLM). The clinician has also received training in MI from Sheffield Hallam University. Additionally, the clinician has over 8 years of clinical experience working in the cardiology department.

### Sampling frame

About seven acute coronary syndrome (ACS) patients are admitted to the acute general hospital per week, including unstable angina, non-ST and ST-segment elevation ACS. About 40% will present with premature ACS [4]. Maltese individuals who are 30 years and older often have quite extensive families. Across 52 weeks (1 year), logistically, if every patient informs their relatives, this would amount to over 100 eligible participants. Since there are no records concerning the recruitment uptake and attrition rate, we will dedicate two years for recruitment and data collection. A sample of 100 participants should be adequate to prove that the process is viable. As the pilot study is not expected to be powered to detect differences between groups, there is no universally accepted calculation for the pilot study sample size [38]. If a meaningful group difference is unknown and the pilot study is intended to establish an effect size for sample size calculation, it is recommended to have 30 to 40 participants recruited in each group. Recommendations for feasibility studies propose a minimum of 30 participants per arm to estimate parameters for future sample size calculations [39, 40]. Ultimately, the sample size decision for a pilot RCT must also consider the research timeline, human resources and costs, and the research objectives [40, 41].

### Procedures

#### Recruitment

**Fig. 1:**
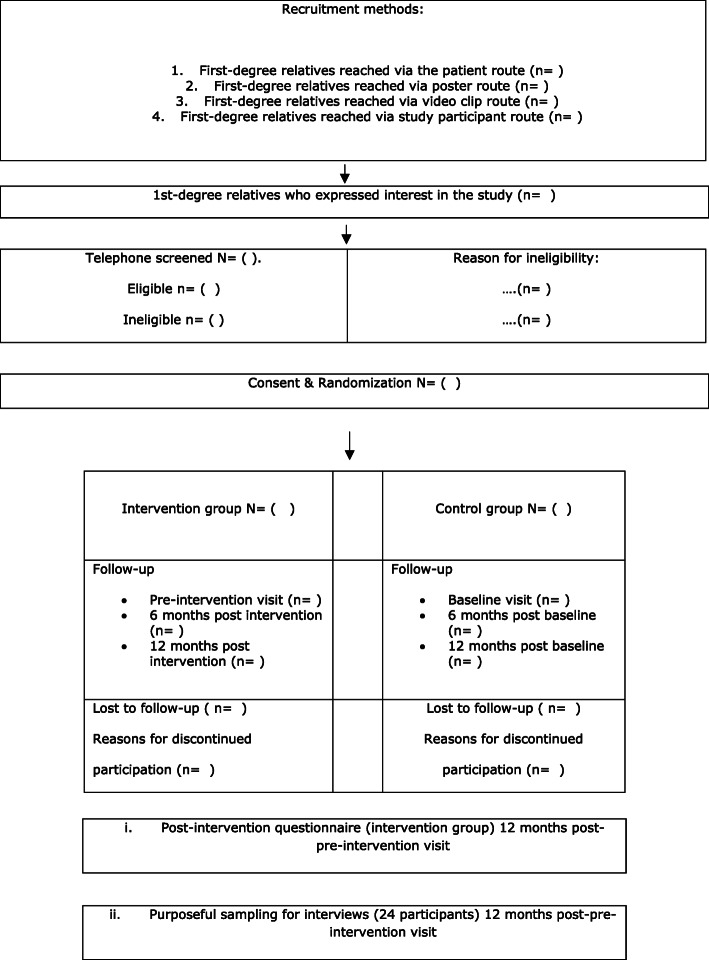
Spirit (Standard Protocol Items: Recommendations for Interventional Trials) flow diagram

The flowchart displayed in Fig. 1 summarises the study design and participant flow, from enrolment to the final follow-up. Before discharge, all patients with acute coronary syndrome are enrolled in a hospital-based cardiac rehabilitation program. Patients with premature CHD, who attend cardiac rehabilitation, will be informed by their nurse about the study, which concerns their relatives. Patients will be the source of help to recruit study participants. Poster promotion of the study will be available at the cardiac rehabilitation reception area and local pharmacies. Poster and video clip promoting the study will be shared via the University's social media (Facebook, News point). Those having a sibling or a parent with premature CHD and are seeking cardiovascular risk assessment will be asked to answer a few questions via telephone to assess if they meet the inclusion criteria. Those found to be eligible will be invited for their baseline assessment at the hospital outpatient clinic.

#### Recruitment method for interviews

All the intervention participants will be asked to complete a questionnaire at the end of the programme.

From a sample of first-degree relatives who completed the programme, 24 individuals will be asked to participate in the interviews. A stratified purposeful sample will be chosen varying on preselected parameters deemed analytically relevant to the Intervention, providing maximum variation. These are sex, age, and risk profile at 12 months. Participants for whom post-intervention outcomes did not improve and participants who had improved their risk profile at 12 months will be purposively selected for focused analysis to identify the Intervention's strengths and weaknesses [32].

#### Group randomisation

Before the baseline/pre-intervention evaluation, approximately 100 participants shall be randomly allocated to one of the two groups:

1. The intervention group shall consist of 50 participants. Participants in this group will have one face-to-face session (evaluation of modifiable risk factors, 2-way risk factor communication, education, and individualised counselling to set up a cardio-protective documented plan) followed by weekly telephone-based cardio-protective reinforcement sessions for up to 12 weeks.
2. The control group shall consist of 50 participants. Participants will have one face-to-face session of verbal lifestyle advice only.

Block randomisation will be applied to balance out the sample size between the intervention and control groups. An opaque sealed envelope technique, using block randomisation with blocks of size four, will be implemented for this study. This method will allow an equal chance of being allocated to one of the two groups and ensure that near-equal numbers are assigned to each group, minimising the risk of group imbalances and allocation bias.

#### Blinding and allocation concealment

Awareness of being part of the intervention group may result in participants behaving differently to what they otherwise might do. Group awareness could have implications for the study's internal and external validity. The participants were informed about the aim of the study; however, not about to which group they have been allocated. Furthermore, participants will be allocated by an independent person from the study, and allocation will occur before the baseline/pre-intervention risk evaluation. Due to resource limitations, the study investigator will deliver the Intervention and assess the outcomes, therefore making part of the blinding impossible. However, blinding will be imposed on the data analyst.

#### Intervention and control arm

All participants will receive the intervention/control in 2–3 days of completing their baseline/pre-intervention assessment. This period will allow time for blood investigation results to be reported on the electronic health records.

##### Intervention arm

**Fig. 2:**
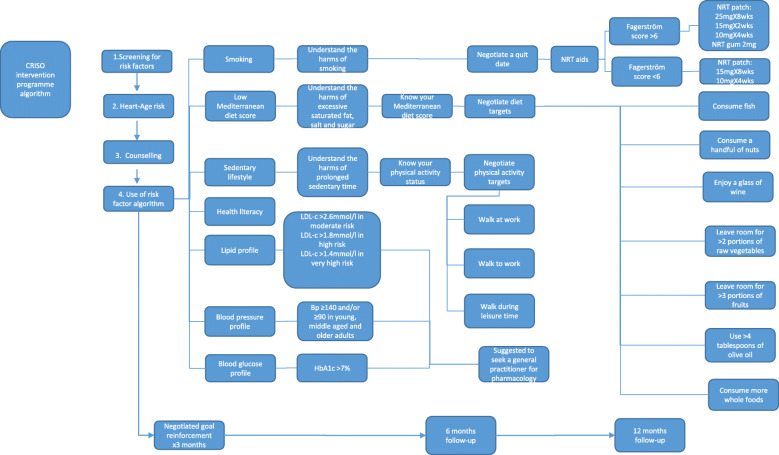
CRISO intervention programme algorithm

Table 1 shows the intervention content and the behaviour change techniques (BCTs) [42] used in the CRISO intervention. Delivery of the CRISO intervention is personalised to the individuals' specific needs. It allows for flexibility across different participants to maximise support in improving their lifestyle behaviours. This would be achieved through modifiable risk factor evaluation, personalised risk communication, and education using Heart Age. A counselling style approach that draws upon some but not all MI principles and practices will be applied. The participant will be asked to reflect and elaborate on their risk profile. This will include the identification and selection of risk factors that the participant would like to modify. A target goal with specific actions to accomplish will be developed with the clinician's support. Figure 2 provides a central illustration of the program. The algorithm will support first-degree relatives more systematically and equip them with a set of specific actions that they can take to reach the target goal. Table 2 gives a greater focus on the use of specific actions. The clinician will also ask the participant to identify a person who can motivate them to implement specific actions. Reflection on obstacles to change and ways to overcome such challenges are also taken into consideration. Summarising will occur at the end of the session, where the clinician will highlight the target goal and the specific actions selected to reach the goal. This will demonstrate that the clinician was actively listening and listening to understand and not to respond. The clinician will support the participant's strengths to increase their confidence in making change. At the end of the session, an electronic preventive cardiology report (Table 3) will be forwarded to the participant. The report will include a cardioprotective plan developed by the participant and the clinician through discussion. The report is to be updated for the participant at 6 months and 12 months follow-up (Table 3).

**Table 1:**
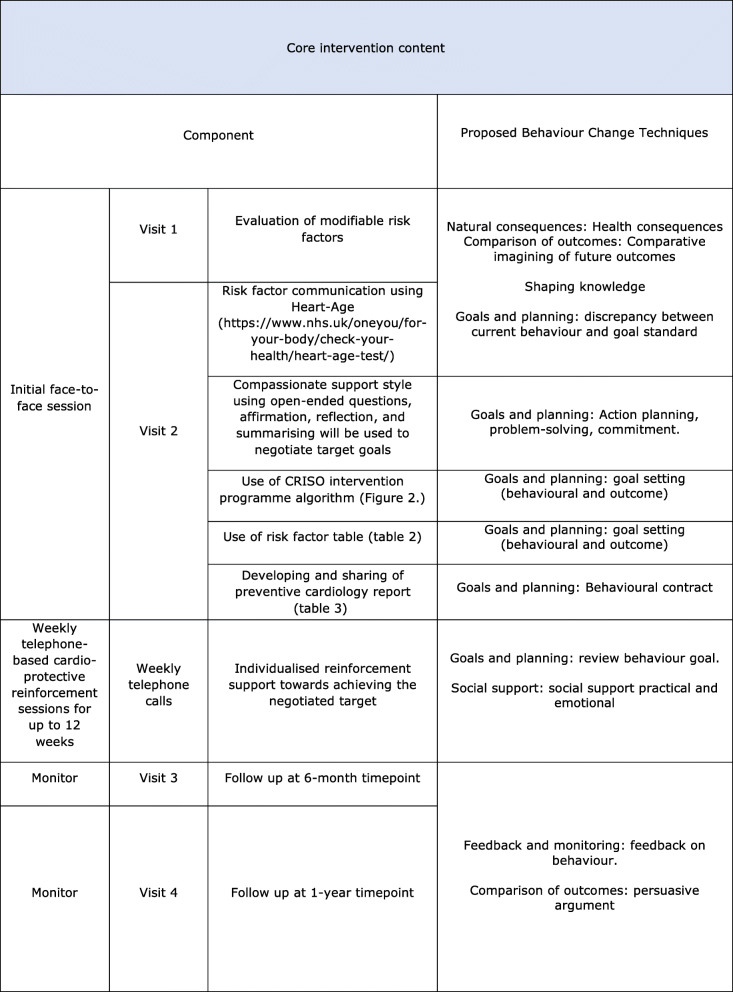
Content summary for the CRISO intervention and BCTs

**Table 2:**
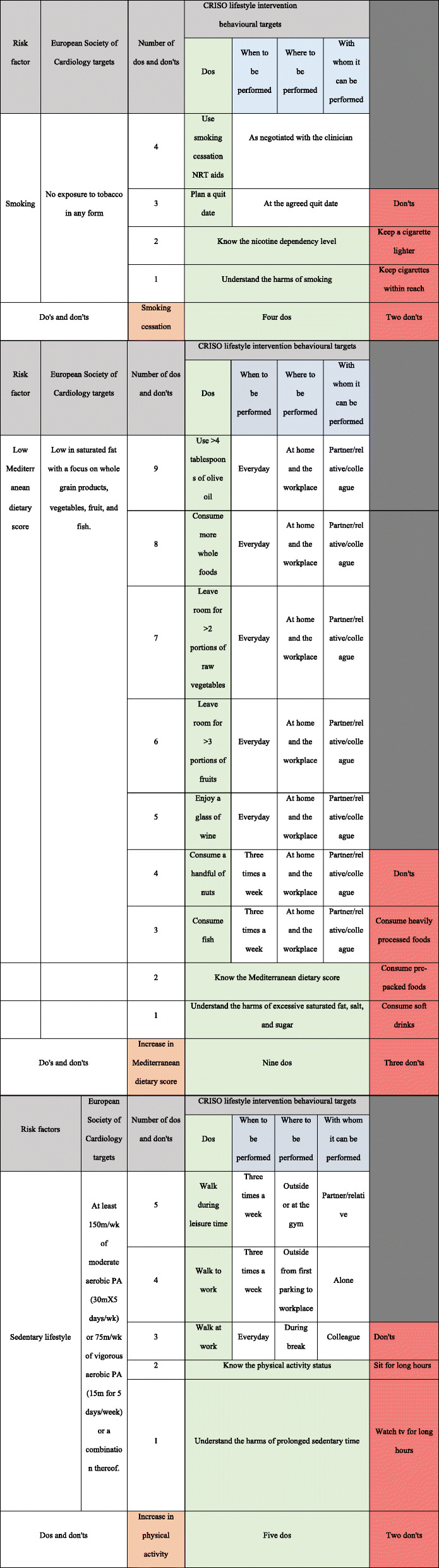
Intervention risk factor and behavioural targets

**Table 3:**
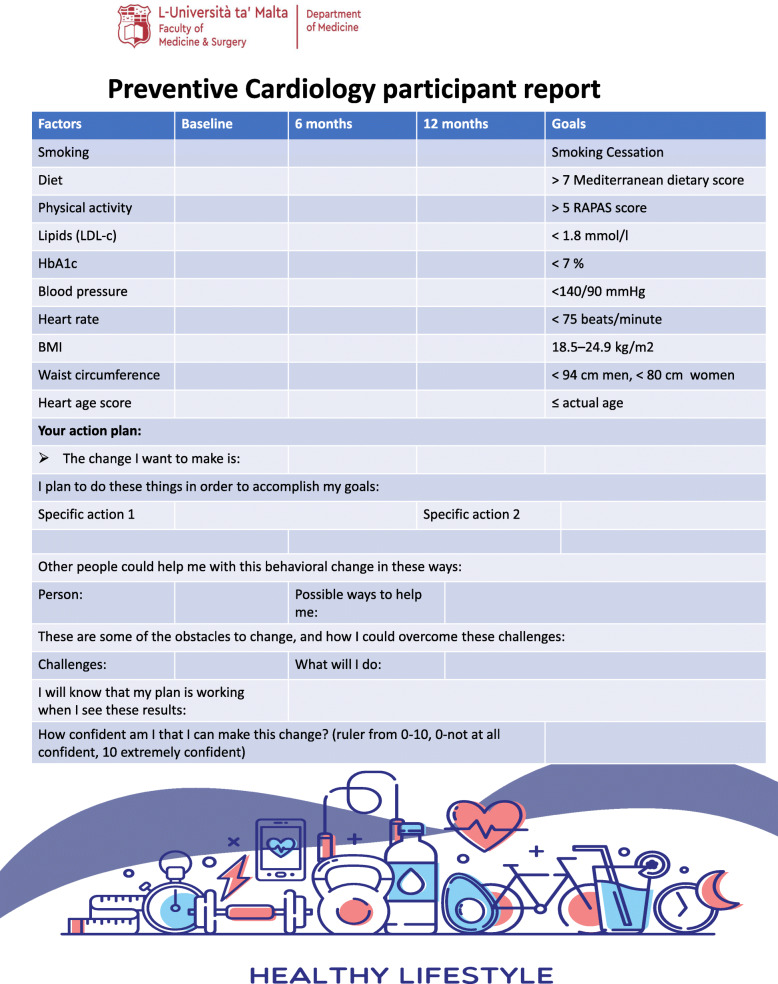
Preventive cardiology report template

**Table 4:**
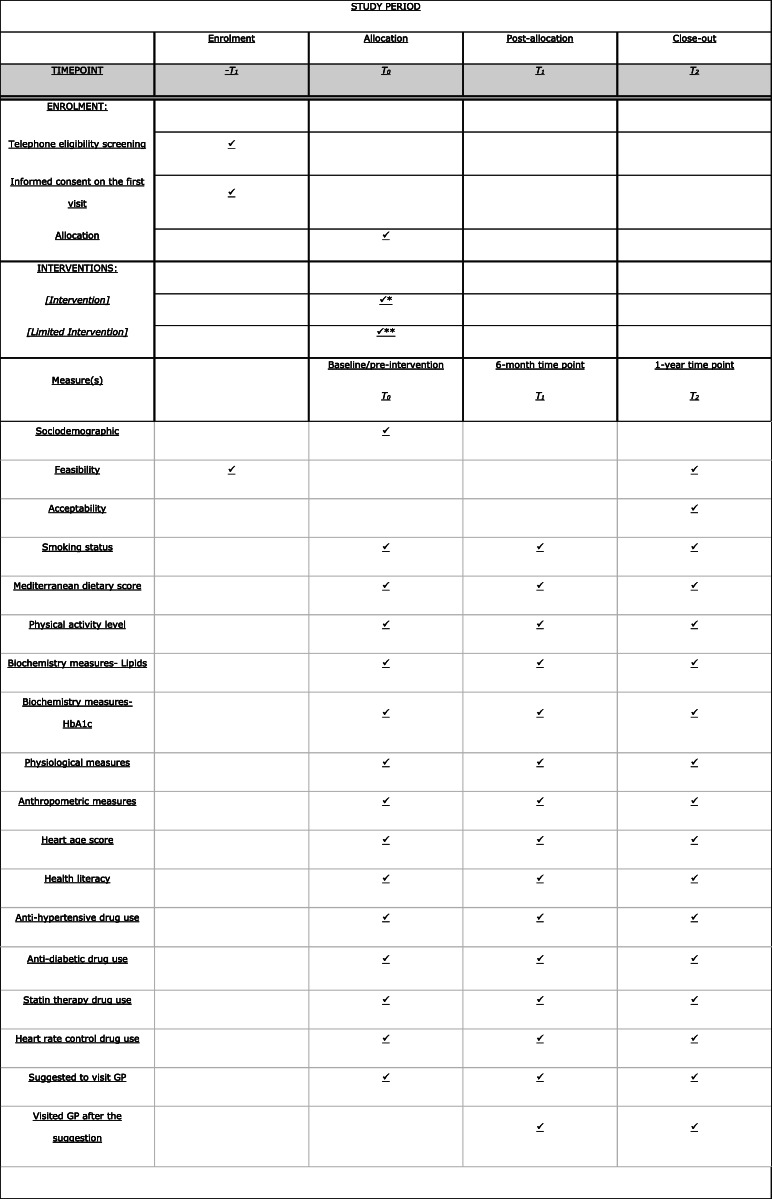
Data collection points

✓* Intervention delivered 2-3 days after T0 for blood results to be issued

✓** Control delivered 2-3 days after T0 for blood results to be issued

Telephone reinforcement sessions following the combined risk communication and counselling session will also be used. In total, participants receive one in-person session and 12 telephone calls (occurring between weeks 1 and 12).

##### Theoretical model

The chosen theoretical model of behaviour change is the Capability Opportunity Motivation–Behaviour model (COM-B). Other theoretical frameworks, such as the behavioural choice theory or theory of planned behaviour, social cognitive theory, and self-regulation theory, appeared ineffective in similar studies [29]. The behaviour change wheel is a theoretical framework that led to the development of the COM-B model. The COM-B is a simple, cost-effective model to apply to all behaviours [43–45]. The model is pivotal in simplifying how behaviours could be targeted [46] and aids in supporting change in one or more of the following: capability, opportunity, and motivation relating to the behaviour itself or behaviours that compete with or support it. Drafting the programme using the COM-B model allowed for insights into how the programme intervention could alter risky behaviours. The COM-B model theory states that there must be the ‘capability’ for modification to modify behaviour (sedentary lifestyle, unhealthy dietary eating patterns, and smoking). First-degree relatives must have the physical and psychological capability to modify risk by developing knowledge through professional contact, discussing risk via visual aids, and discussing risk-reduction methods through the provision of feedback and monitoring. The CRISO program may facilitate an environment for opportunistic screening, improve professional contact, and encourage first-degree relatives to seek risk assessment. The Heart Age risk calculator was identified to educate about, evaluate, and communicate risk to increase understanding of risk and support participants to act on it. In return, this should improve understanding of risk and enhance the capability and opportunity to modify risk factors. Using a counselling style adapted from MI might further support individuals in identifying and select behaviours that require modification [29].

##### Control arm

About 70% of general practitioners' CVD risk consultations happen in verbal terminology [47]; participants assigned to the control arm will only receive one in-person session based on verbal lifestyle advice, referring to the European Society of Cardiology lifestyle targets about smoking, dietary pattern, and physical activity levels [3].

#### Follow-up

Participants will be assessed at the same time points after being recruited in the study. Follow-up points for study participants translate into the following phases: baseline/pre-intervention, 6 months, and 12 months after baseline/pre-intervention. Table 4 shows data collection points.

### Data analysis

#### Descriptive summary of sample data

A description of participants' characteristics, including demographic information and baseline measurements, will be presented for both groups.

The entire cohort will also be summarised descriptively. For categorical data (smoking), frequencies and valid proportions and percentages will be used [48]. Numerical variables will be summarised using mean and standard deviation, or median and range or interquartile range. Risk ratios will be generated for selected outcome variables, dichotomised as necessary, from proportions of participants meeting particular criteria (e.g. control of blood pressure) in the control and intervention groups.

#### Primary outcome analysis (Quantitative and Qualitative data)

##### Objective 1: Feasibility

The proportion of those who showed interest in the study and are eligible to participate, agreed to participate, and those who remained in the study will be recorded. Percentages (%) of the number approached for participation will be used [48]. The average time to deliver the Intervention will be measured.

##### Objective 2: Acceptability

A post-intervention questionnaire, consisting of ruler scales (0–10), was developed to rank the intervention components' order. This data will be collected from all the intervention participants.

The audio-recorded data from the interviews shall be transcribed verbatim by the author. The data will be validated with the study participants and analysed using a thematic analysis method [49]. The phases to be applied are familiarisation with the data and identifying items of interest, code generation, management of the coding process, reviewing potential themes, defining and labelling the themes, and producing a report [49].

#### Secondary outcome analysis

##### Objective 3: Estimates of effect and preliminary evidence of efficacy

The likely range of effects will be estimated using liberal confidence intervals. Preliminary estimates of intervention efficacy will be established via hypothesis tests with liberal alpha values (e.g. 0.15, 0.2). Numerical outcomes measured on an interval level, and numerical outcomes measured on an ordinal scale that can be considered approximate to interval level, will be assessed using independent samples t tests. Categorical data will be assessed using the chi-squared test for association.

Intention-to-treat analysis will be used to test for the differences between the two groups at the endpoint [50]. Intention-to-treat analysis is considered the gold standard method for evaluating a new intervention. This method includes all study participants whether or not they have completed the study program and will preserve the randomisation process as it keeps the groups balanced in their number of participants in case of any drop-outs, thus minimising biases [51].

Risk ratios (RRs) will be generated for selected outcome variables, dichotomised as necessary, from proportions of participants meeting particular criteria (e.g. control of blood pressure) in the control and intervention groups.

Effect sizes such as Cohen's d statistics will be calculated for each outcome measure and presented alongside parameter estimates in each group and associated confidence intervals. These, in conjunction with other statistics derived from the pilot analysis, may be used to estimate sample size for a subsequent follow-up RCT [38].

##### Objective 4: Preliminary indications of associations

The preliminary indications of associations between outcome variables will be facilitated using correlation and association measures (Pearson's product–moment correlation coefficient for numerical outcomes) (uncorrected chi-squared test for association and/or Fisher's exact test for categorical outcomes). Liberal confidence intervals (e.g. 80%, 90%) will be applied.

### Ethics

A detailed information letter will be provided to each participant. This letter will explain the nature of the study and what participation would involve. After the participant is given time to clarify anything about study participation, the principal researcher will obtain each participant's consent. It will be made clear that data will be retained in an anonymous form. Participants are free to withdraw at any time during the study. Data will be safely stored in an encrypted computer, and only the principal researcher will have access to this data. This is per data protection directives (GDPR EU 2016/679). The protocol was approved by the University of Malta Ethics Committee (UNIQUE FORM ID: 3756_191119) and the Malta Health Ethics Committee (HEC03 CT01/20).

## Discussion

This study aims to evaluate a pilot study of a preventive cardiology program designed to promote risk reduction in a potential risk group. Issues of feasibility in the study design need to be tested before conducting a larger-scale randomised controlled trial [52]. The testing requires determining acceptability, estimating participants' recruitment and retention rate, evaluating the Intervention's operational strategy, and estimating the sample size needed for a full-scale study. The Medical Research Council framework guidance reports that a pilot study must be a scale model of the planned full-scale Intervention and must identify and address any issues [52].

**Fig. 3:**
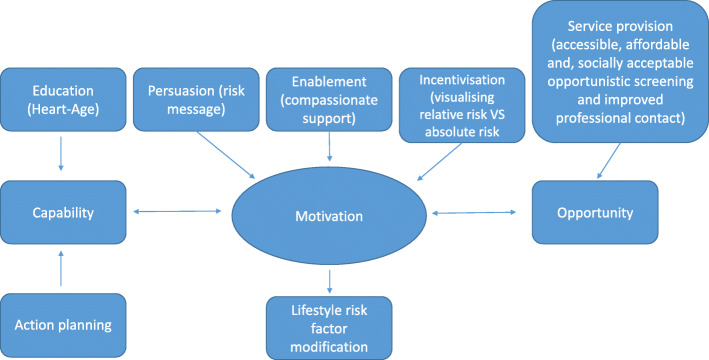
Application of COM-B in the CRISO program to modify risk

Having access to a cardiovascular risk assessment (Opportunity) and being able to understand heart risk and ways to change risk (Capability) might increase motivation to modify present risks (Fig. 3). The key determinant is motivation; however, motivation alone is not sufficient as individuals still need to understand their risk, develop the skills required to reduce it, and have good access to cardiovascular risk assessment and follow-up. In this way, the interaction between Capability, Opportunity, and Motivation could be pivotal in supporting and bringing about the desired behaviour changes [43]. Figure 3 shows the application of COM-B in the CRISO program to modify risk. This approach was adopted from Michie et al. [53].

### Strengths and limitations

The Intervention's delivery and all measurements will be performed by the same researcher, producing more consistency. A guide was developed to support the fidelity of the Intervention. A randomly chosen set of 10 audio-recorded sessions will be assessed against an intervention guide. A randomised controlled design was selected as it would allow testing for potential intervention effectiveness. This can provide supportive data for preventive program development to be tested on a larger sample future RCT. Part of the study will include conducting interviews, which will help us understand participants' views about the Intervention's implementation and acceptability. This data will allow valuable information on what components are useful to deliver the intervention and support risk factor modification in potential risk groups. The internal pilot phase will dictate any modifications needed in the intervention. However, the data collected for this purpose is proposed in the final efficacy analysis, disregarding that part of the data which came from a pilot phase.

### Future research opportunities

The CRISO study has considered the possibility of future studies. If this study were to establish feasibility, a large scale, randomised controlled trial will be designed and implemented, with the possibility to facilitate the assessment of countrywide effects. This study would be the first in Malta to test the effectiveness of an evidence-informed intervention to support first-degree relatives to modify risk.

As the study is to recruit first-degree relatives, these would generally have a combination of clinical, biochemical, and genetic risk factors [54, 55]. In the CRISO study, intervention participants who are found to have alarming parameters (atherogenic lipid profile, obesity) will be asked to consent for blood genetic testing at a 1-year time point. Having such samples will open another route for future research to determine if there is an association between genetic risk score (a combination of variants) in those with alarming parameter/s (phenotypes—traditional risk factors). If an association is found, this may suggest that conventional risk should be combined with genetic risk, which may give the total potential risk. This study might further refine preventive cardiology.

### Counterbalancing for confounding factors and bias

There are several possible interacting variables: age, health literacy, educational levels, isolation, employment, sex, psychometric scales, time of the cardiac patient event, and family clustering effects. These multiple variables make it rather complex to decide on stratified sampling. Additionally, as the sample solely depends on the recruitment process, an element that still needs to be tested, it was decided not to stratify at this phase.

The chosen method to generate the allocation sequence should produce comparable groups. However, due to a lack of resources, allocation concealment is not possible. This study is single-blinded, where participants will only be informed about the aim of the study but not about being part of an intervention or control group. However, the researcher will be aware of which participants have been allocated to the intervention group. Thus, there is a risk of performance and detection bias. An independent person from the study will perform group allocation. Also, group allocation will take place before the baseline risk assessment. Using these methods will avoid any attempt to allocate participants to the Intervention according to their risk profile. This step is necessary when the study's nature does not allow proper blinding [56].

Numbers of the total sample randomised, those lost to follow-up, and reasons for withdrawal will be recorded for each group. The risk of attrition bias will be minimised by carrying forward the last observation. This method will keep the groups balanced if there is a loss to follow-up/missed measuring time points.

## Conclusions

This research will provide new knowledge about the feasibility of the processes and inform the intervention components' acceptability by siblings and offspring. The Intervention may provide a new preventative approach to support individuals to modify their risk behaviours.

## Study status

The study started recruiting in September 2020.

## Data Availability

The anonymised participant datasets generated and/or analysed during the current study will be made available in the University of Malta institutional open access data repository https://www.um.edu.mt/library/oar/.

## Abbreviations

ASCVD: Atherosclerotic cardiovascular disease
CHD: Coronary heart disease
CVD: Cardiovascular diseases
LDL-c: Low-density lipoprotein cholesterol
MEDAS: Mediterranean diet adherence screener
RAPA: Rapid assessment of physical activity
CDC: Centres for Disease Control and Prevention
HLQ: Health literacy questionnaire
ACS: Acute coronary syndrome
COM-B: Capability Opportunity Motivation–Behaviour
